# Immune checkpoint alterations and their blockade in COVID-19 patients

**DOI:** 10.1097/BS9.0000000000000132

**Published:** 2022-08-01

**Authors:** Jiaxiong Tan, Yangqiu Li

**Affiliations:** a Department of Hematology, First Affiliated Hospital, Jinan University, Guangzhou, China; b Institute of Hematology, School of Medicine, Key Laboratory for Regenerative Medicine of Ministry of Education, Jinan University, Guangzhou, China

## Abstract

Coronavirus disease 2019 (COVID-19) is a highly contagious disease that seriously affects people’s lives. Immune dysfunction, which is characterized by abnormal expression of multiple immune checkpoint proteins (ICs) on immune cells, is associated with progression and poor prognosis for tumors and chronic infections. Immunotherapy targeting ICs has been well established in modulating immune function and improving clinical outcome for solid tumors and hematological malignancies. The role of ICs in different populations or COVID-19 stages and the impact of IC blockade remains unclear. In this review, we summarized current studies of alterations in ICs in COVID-19 to better understand immune changes and provide strategies for treating COVID-19 patients, particularly those with cancer.

## 1. INTRODUCTION

Coronavirus disease 2019 (COVID-19) is a highly contagious disease caused by severe acute respiratory syndrome coronavirus 2 (SARS-CoV-2) infection, which can lead to an excessive inflammatory reaction and acute lung injury, leading to respiratory failure or death.^1^ Studies have shown that immune dysfunction, such as imbalance in the lymphocyte proportion, and the absolute decrease in T cells, which is caused by excessive apoptosis, are important characteristics of severe patients with poor prognosis.^2–4^ Recently, numerous studies have demonstrated that ICs, which are expressed on immune cells, play an important role in cancer immunosuppression and infectious diseases.^5^ The dysfunction in immune cells in COVID-19 may be associated with alterations in ICs.

As a “brake” for suppressing the immune system when the body is invaded by tumor antigens or pathogens for a long period of time, immune cells, particularly T/NK cells, pathologically express key ICs such as programmed death 1 (PD-1), cytotoxic T lymphocyte-associated antigen-4 (CTLA-4) and T cell immunoglobin and mucin domain 3 (Tim-3).^6–8^ When these ICs bind to specific ligands, T cells enter a state of impotent exhaustion, thus inhibiting immune surveillance and clearance.^9^ IC blockade (ICB) have been widely studied and applied in many types of tumors, such as melanoma, non-small cell lung cancer, lymphoma, and other infectious diseases including hepatitis B virus, human immunodeficiency virus, and cytomegalovirus, and have been shown to significantly improving clinical outcome.^10,11^

**Figure 1.**
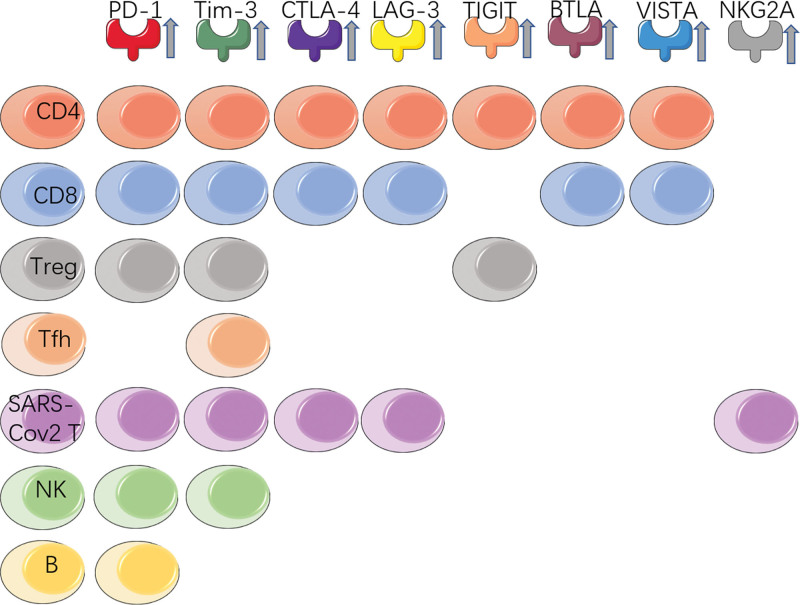
Summary of the increased expression of immunosuppressive receptors on immune cells.

Recently, increasing data have shown that up-regulation of ICs results in dysfunction in NK cells, monocytes, and B cells in patients with COVID-19 (Table 1, Figure 1)^2,3^ and an increase in specific or soluble ligands in tissue endothelium, antigen-presenting cells (APCs), and plasma (Table 2).^12^ However, the expression levels of the ICs can be maintained mostly at normal levels in mild non-hospitalized COVID-19 and convalescent patients.^49^ While, there are also reports indicating that the expression of major co-inhibitory receptors including PD-1, CTLA-4, and T cell immunoglobulin and ITIM domain (TIGIT) is not detected in samples from COVID-19 patients.^3,21,25^ In addition, it has been reported that the use of anti-PD-1/PD-L1 alone or in combination with anti-CTLA-4 immunotherapy increases the risk of overlapping lung injury and co-morbid mechanisms for COVID-19 in cancer patients.^50^ Here, we summarized recent research on the distribution characteristics and alterations of ICs in COVID-19 patients and discuss the role and clinical significance of ICB in disease control.

**Table 1:** The distribution of immune checkpoints in patients with COVID-19.

ICs	Expression	Cell type/fluid	Performance	Sample style	Sample size (n)	Detection method	Area	Reference
PD-1	Up	NK and T cells	Death	Lung, kidney	11	DSP, mIHC	China	^1^
	Up	Plasma	Severe case	Plasma	109	FCM	China	^12^
	Up	CD4+ and CD8+ T cells	Severe case	PB	100	FCM	Iran	^13^
				PB	522	FCM	China	^14^
				PBMC	76	FCM	China	^15^
	Up	CD8+T cells	Elderly mild cases	PB	42	FCM	Italy	^16^
				PB	30	FCM	Germany	^17^
	Up	CXCR3+CD4+ and CCR6+CD8+ T cells	Severe cases	BALF/PB	22(PB), 9(BALF)	FCM	Netherlands	^18^
	Up	CD57+T and CD38+TCM cells	Unknown	PB	39	FCM	Italy	^19^
	Up	Treg cells	Unknown	PB	39	FCM	Italy	^19^
	Up	CD56dim NK cells	Unknown	PB	35	FCM	Italy	^20^
	Down	PB cells	Severe cases	PB	14	RNA-seq	China	^3^
				PBMC	33	CITE-seq	USA	^21^
	Up	Memory T cells and CD38+SARS-CoV-2–specific T cells	Acute severe cases	PB	206	FCM	Sweden	^22^
	Up	SARS-CoV-2–specific T cells	Acute and convalescent cases	PB	235	FCM	Korea	^23^
	Up	B cells	Unknown	PB	20	FCM	Germany	^24^
				PB	85	FCM	USA	^25^
Tim-3	Up	Memory T cells and SARS-CoV-2–specific T cells	Acute severe cases	PB	206	FCM	Sweden	^22^
	Up	SARS-CoV-2-reactive CD8+ T cells	Unknown	PB	39	scRNA-seq	UK	^26^
	Up	Plasma	Severe cases	Serum	24	ELISA	China	^27^
				Plasma	39	EIA	Norway	^28^
				Plasma	55	ELISA	China	^29^
	Up	NK and CD8+ T cells	Severe cases	PB	32	FCM	Italy	^30^
				PBMC	44	FCM	Iran	^31^
	Up	NK and T cells	Death	Lung, Kidney	11	DSP, mIHC	China	^1^
	Up	CD4+ and CD8+ T cells in	Severe cases	PBMC	76	FCM	China	^15^
				PBMC	20	FCM	UK	^2^
		CD4+ T cells		PBMC	44	FCM	Iran	^32^
		Tfh cells		PB	11	FCM	China	^33^
	Up	CD3+ T cells	Acute severe cases	PBMC	92	FCM	Germany	^34^
	Up	Treg cells	Convalescent cases	PBMC	30	FCM	China	^35^
	Up	CD4+ and CD8+ T cells	Unknown	Skin	8	IHC	Italy	^36^
CTLA-4	Down	PB cells	Severe cases	PB	14	RNA-seq	China	^3^
	Up	SARS-CoV-2–specific T cells	Severe cases	PB	108	FCM	Canada	^37^
	Up	Memory T cells and SARS-CoV-2–specific T cells	Acute severe cases	PB	206	FCM	Sweden	^22^
	Up	CD4+ and CD8+ T cells	Severe ARDS cases	PB/BALF	4	FCM	UK	^38^
	Up	Plasma	Severe cases	Plasma	109	FCM	China	^12^
LAG-3	Up	Memory T cells and SARS-CoV-2–specific T cells	Acute severe cases	PB	206	FCM	Sweden	^22^
	Up	SARS-CoV-2-reactive CD8+ T cells	Unknown	PB	39	scRNA-seq	UK	^26^
	Up	CD4+ and CD38+ HLA-DR+CD8+ T cells	Severe cases	PBMC	10	scRNA-seq, FCM	USA	^39^
	Up	CD4+ and CD8+ T cells	Unknown	PBMC	20	FCM	UK	^2^
TIGIT	Down	SARS-CoV-2–specific T cells	Unknown	PB	206	FCM	Sweden	^22^
				PB	108	FCM	Canada	^37^
	Down	CD4+ and CD8+ T cells	Unknown	PB	50	FCM	Germany	^40^
				PBMC	20	FCM	UK	^2^
	Down	NK cells	Unknown	PB	50	FCM	Germany	^40^
	Down	CD4+ and CD8+T cells	Non-ICU cases	PB	144	FCM	Spain	^41^
	Up	CD4+ T cells	Acute cases	PB	85	FCM	USA	^25^
		Treg and CD4+TM cells		PB	50	FCM	Germany	^40^
				PBMC	20	FCM	UK	^2^
BTLA	Up	CD8+TM and TEM cells	Unknown	PBMC	20	FCM	UK	^2^
	Up	CD4+ and CD8+ T cells	Active cases	NPSs	430	RNA-seq	United Arab Emirates	^42^
				Lung/BALF	16	RNA-seq		
	Up	Plasma	Severe cases	Plasma	109	FCM	China	^12^
	Up	CD4+ and CD8+ T cells	Active cases	PB	50	FCM	Germany	^40^
VISTA	Up	CD154+CD4+ and CD137+CD8+T cells	Unknown	PB	108	FCM	Canada	^37^
	Up	CD4+ and CD8+T cells	Mild cases	PBMC	45	FCM	USA	^43^
	Up	CD4+ and CD8+T cells	Unknown	PBMC	108	FCM	Canada	^37^
NKG2A	Up	SARS-CoV-2–specific T cells	Severe cases	PB	108	FCM	Canada	^37^

ARDS = acute respiratory distress syndrome, BALF = bronchoalveolar lavage fluid, CITE-seq = cellular indexing of transcriptomes and epitopes by sequencing, DSP = digital spatial profiling, EIA = enzyme immunoassays, ELISA = enzyme-linked immunosorbent assay, FCM = flow cytometer, mIHC = multiplex-immunohistochemistry, NPSs = nasopharyngeal swabs, PB = peripheral blood, PBMC = peripheral blood mononuclear cell, RNA-seq = RNA sequencing, scRNA-seq = single-cell RNA sequencing, TCM = central memory T cells, TEM = effector memory T cells, Tfh = follicular helper T cell, TM = transitional memory T cells, UK = United Kingdom, USA = United States of America.

**Table 2:** Distribution of immune checkpoint ligands in COVID-19.

IC ligands	Expression	Cell type/fluid	Performance	Sample style	Sample size (n)	Detection method	Area	Reference
PD-L1	Up	MON and DCs	Severe cases	PBMC	19	FCM	Czech Republic	^44^
				PB	20	FCM	Portugal	^45^
Gal-1, -3	Up	Plasma	Severe cases	Plasma	84	ELISA	Turkey	^46^
Gal-3, -9	Up	Plasma	Severe cases	Plasma	55	ELISA	China	^29^
Gal-9	Up	HLA-DR+ and CD80+ monocyte	Unknown	PB	146	FCM	Canada	^47^
	Up	Neutrophils	Unknown	PB	146	FCM	Canada	^47^
CD155	Up	Plasma and pulmonary epithelial cells	Severe cases	Serum/lung	46	Proteomics/IHC	China	^48^

DCs = dendritic cells, ELISA = enzyme-linked immunosorbent assay, FCM = flow cytometer, Gal = galectin, IC = immune checkpoint, IHC = immunohistochemistry, MON = monocytes, PB = peripheral blood, PBMC = peripheral blood mononuclear cell, PD-L1 = programmed death-ligand 1.

## 2. PD-1

PD-1 is a co-inhibitory receptor expressed on a variety of immune cells.^7^ After the TCR recognizes an antigen, it forms a specific cross-link with the ligand PD-L1\PD-L2, which plays a negative regulatory role.^7,51^ Based on the effectiveness of a monoclonal antibody directed against the PD-1/PD-L1 axis in reversing T cell function,^10^ the role of anti-PD-1 therapy in the clearance of SARS-CoV-2 was investigated.^52^

With regards to the relationship between ICs and COVID-19, the earliest research came from pathological examination data from autopsies of COVID-19 patients. It was found that there was an abnormal distribution of T cells in lung, kidney, and bronchoalveolar lavage fluid (BALF). PD-1 and Tim-3 were the signs of severe immunosuppression in COVID-19, and they were mainly correlated with males and the elderly.^1,18^ Based on the theory that soluble checkpoint molecules are produced by membrane-bound protein cleavage or mRNA expression, Kong Y et al detected 13 soluble co-inhibitory molecules, including sPD-1 and sPD-L1, in plasma samples of severe or critical patients, and these were significantly higher than that in mild and asymptomatic infected patients, and they maintained a continuous increasing trend upon dynamic analysis, with the exception of PD-L2.^12^ Correspondingly, a series of descriptive reports demonstrated that PD-1 was up-regulated in T cells, NK cells, and B cells from the PB of COVID-19 patients.^20,24^ Subgroup studies of PB samples almost consistently concluded that PD-1 expression was higher and immunosuppression was more severe in critically ill patients, hospitalized patients, and the elderly, particularly those over 80 years old.^14,17,53^ While, the percentage of PD-1+CD4+ and PD-1+CD8+ T cells decreased gradually after treatment.^49^ However, some studies have not found a difference in PD-1 and PD-L1 expression and CD8+ T cell dysfunction.^3,21,54^ The reason for the difference in reports may be due to ignoring the distinction in immune cell function. Not all T cells are virus-specific immune cells, and there is a lack of data supporting effector function.

De Biasi S et al found that PD-1 was mainly overexpressed on CD38+ central memory T cells, effector memory T cells, and primitive regulatory T (Treg) cells in the COVID-19 activated phenotype, but there was no difference in the expression levels of the terminal TEM and stem cell memory T cells subsets.^19^ Saris A et al pointed out that the high expression of PD-1 in CXCR3+CD4+ and CCR6+CD8+ T cells was the reason for the decrease in the T_H_1 cell proportion and insufficient secretion of granzyme and perforin by cytotoxic CD8+ T cells (CTLs) in severe COVID-19 patients.^18^ This result was supported by the finding of up-regulated PD-1, Tim-3, and lymphocyte activation gene 3 (LAG-3) on SARS-CoV-2–specific T cells in the acute stage of COVID-19, and it was negatively correlated with the time after the onset of symptoms.^22^ However, Rha MS et al found that PD-1+, SARS-CoV-2–specific CD8+ T cells could still produce interferon (IFN)-γ in the acute phase and convalescence.^23^ It was suggested that up-regulation of PD-1, CTLA-4, Tim-3, and V-domain Ig suppressor of T cell activation (VISTA) on SARS-CoV-2–specific T cells may be related to the over activation of T cells.^37^ Overall, the findings suggested that transient expression of co-inhibitory receptors on activated T cells prevent a harmful overactive immune response in COVID-19.^55^

## 3. Tim-3

Another important co-inhibitory receptor, Tim-3 was initially found to be expressed in CD4+ type 1 helper T cells (T_H_1 cells) and CTLs.^6^ Subsequently, Tim-3 was detected on Tregs, myeloid cells, NK cells, and tumor cells.^6^ At present, it is considered that blockade of Tim-3 signaling can be used as a supplement to the failure of anti-PD-1/PD-L1 therapy.

Pathologists have found a higher level of Tim-3 in the center of the pulmonary inflammatory storm in cadaver samples of COVID-19 patients, which is consistent with a report from a multi-center, single-cell sequencing study where Tim-3 expression was increased and a high concentration of sTim-3 was detected in the plasma of critically ill patients.^1,28,56^ Interestingly, the proportion of Tim-3+ and PD-1+Tim-3+ cells among T cells and NK cells in male lung tissue is significantly higher than that in females, which is related to a local high viral load, suggesting that high expression of Tim-3 in male immune cells has a poor prognosis.^1^ A case report from Spain confirmed the above findings.^57^ In this report of a couple who live together, close in age, and were infected with the same load of SARS-CoV-2 virus, it was demonstrated that the husband gradually developed severe pneumonia, showed progressive respiratory failure, and became hospitalized on the 10th day after onset. In contrast, his wife only displayed mild to moderate tolerable symptoms. Dynamic data display that the male patient maintained a high proportion of Tim-3+CD4+ and Tim-3+CD8+ T cells throughout the course of the disease, and sGal-9 had a significant concentration difference that was detected in the plasma 2 days before the disease became worse.^57^

A number of studies have reported that Tim-3 was detected in CD4+ and CD8+ T and NK cells in the PB of COVID-19 patients accompanied by immune dysfunction.^32^ Diao B et al found that the expression of the *Tim-3* and *PD-1* genes in T cells gradually increased with the progression of clinical symptoms.^14^ These findings may explain the higher level of Tim-3 detected in PB samples of critically ill COVID-19 patients.^15^ However, a high proportion of Tim-3+ Treg and Tim-3+ CD8 cells was still detected in convalescent patients, but the secretion of granzyme B was limited.^35^ In addition, it is known that CD4+ T cells can regulate the immune response of CTLs through T_H_1 cells and Tregs and activate B cells through T follicular helper (Tfh) cells to promote the production of virus-specific antibodies. In contrast, a recent study found that the decrease in CD4+ T cells in COVID-19 patients was accompanied by up-regulation of Tim-3 in T_H_1, Treg, Tfh cells, and they may reduce the functions described above.^32,33,35^ In addition, Herrmann et al^2^ found the expression of LAG-3 and TIM-3 in all subsets of CD8+ and CD4+ T cells (including naive, CM, TM, EM, EMRA, and SARS-CoV-2–specific T cells) of critical COVID-19 patients increased significantly.

More interestingly, Cazzato et al found that TIM-3 was accompanied by the expression of another ligand, high mobility group box 1(HMGB-1), in CD4+ and CD8+ T cells from skin biopsy samples of COVID-19 patients. Suggesting that in addition to the respiratory tract, the skin, the “sentinel” of the immune system, is also affected.^36^

## 4. CTLA-4

CTLA-4 induces inhibitory signals in regulatory or effector T cells by competitively binding to B-7 ligand, which is an important protective mechanism to prevent a super-immune response. Although higher concentrations of soluble CTLA-4 can be detected in the plasma of critical COVID-19 patients compared to mild and healthy individuals,^12^ CTLA-4 was only slightly expressed on T cells in PB from critical patients.^38^ There was no difference in *CTLA-4* gene expression by RNA-seq in PB samples between COVID-19 patients and healthy controls.^3^ However, a higher proportion of CTLA-4+CD4+ and CTLA-4+CD8+ T cells was detected in BALF compared with the PB of critical COVID-19 patients with acute respiratory distress syndrome.^38^ Overall, it was reported that CTLA-4 was still highly expressed on CD4+ T cells including SARS-CoV-2–specific CD4+T cells 1 year after recovery.^58^

## 5. BTLA

B and T lymphocyte attenuator (BTLA), a co-inhibitory receptor mainly expressed on B and T cells, has been found to have abnormal expression and may be an indicator for late lymphocyte exhaustion. The ligand for BTLA, herpes virus entry mediator was found to play an auxiliary role in immune escape in PD-L1-deficient lung tumors.^59^ Transcriptome data from nasopharyngeal swabs (NPSs) and lung autopsy samples from COVID-19 cases have been published, demonstrating a pattern of upregulation of the mRNA levels of 8 ICs, including *BTLA, LAG-3, CTLA-4*, and *PDCD1* during acute COVID-19.^42^ The above results are consistent with the findings that high concentrations of soluble BTLA can be detected in the plasma of critically ill COVID-19 patients.^12^ Interestingly, 1 report showed that COVID-19 is very similar to malaria infection, and higher levels of BTLA expression can be detected on TM and EM CD8+ T cells.^2^

## 6. TIGIT

TIGIT is an inhibitory molecule that is expressed on NK/T cells, and it was found to be up-regulated to induce T cell exhaustion in leukemia patients. TIGIT mainly competes with DNAM-1 and CD96 for CD155 to induce immunosuppression. Studies have shown high expression of CD155 in SARS-CoV-2 infected cells^48^; however, the findings of TIGIT alteration in COVID-19 remain controversial. Although many studies have found that TIGIT is up-regulated in patients with acute COVID-19, ICU patients, and the deceased, the Treg, CD4+ TM cell, and CD8+ T cell subgroups are up-regulated and recovered in the rehabilitation cohort.^40^ Reports also have shown a decrease in TIGIT in NK cells^2^ and normal TIGIT expression on CTLs from COVID-19 patients regardless of the need for hospitalization.^22,25^

## 7. LAG-3

LAG-3 is a marker for NK cell dysfunction. All CD8+ and CD4+ T cells (including naive, CM, TM, EM, and EMRA) in patients with COVID-19 demonstrated an increase in the expression of LAG-3.^2^ Multi-omic single-cell analysis has also found up-regulation of LAG-3 on most T/NK cells, with the exception of Tregs, in samples from advanced COVID-19 patients.^39^ In addition, it was also found that LAG-3+CD4+ T cells are negatively correlated with activated monocytes. Thus, LAG-3 may play a regulatory role after the activation of immune cells.^39,60^

## 8. VISTA

VISTA is an immune checkpoint protein with suppressive effects on CD4+ and CD8+ T cells.^61^ In chronic viral diseases (including chronic SARS-CoV-2 infection), exhausted T cells with high expression of VISTA may appear.^19^ Rendeiro et al found that VISTA was significantly up-regulated in CD4+ and CD8+ T cells in mild and moderate COVID-19 patients. Interestingly, the frequency of VISTA expression in severe patients was decreased, but it was still higher than that in healthy controls and the convalescent.^43^ In addition, a study from Canada also found that the SARS-CoV-2–specific T cells (CD154+CD4+, CD137+CD8+ T cells) stimulated by antigens significantly increased VISTA molecules.^37^ Recent studies of VISTA on T cells, monocytes, and myeloid cells have highlighted its potential significance in driving cytokine storms after viral infection.^62^

## 9. OTHER IMMUNOSUPPRESSIVE RECEPTORS

CD47 is a glycoprotein widely expressed in the immunoglobulin superfamily. CD47 interacts with signal regulatory protein α (SIRP α) to transmit an anti-phagocytic “don’t eat me” signal to mediate immune escape.^63^ The *CD47* gene is up-regulated in human bronchial epithelial A549 lung cancer cells, Caco2 colorectal carcinoma cells, and Calu-3 lung cancer cells infected with SARS-CoV-2 accompanied by an increase in SIRPα in monocyte infected cells.^64^ It was found that CD47 may be related to COVID-19 pulmonary fibrosis, which can be reversed by an anti-CD47 antibody in a mouse model study.^65,66^

NKG2A, identified as another novel IC protein, was also found to be overexpressed on CTLs and NK cells in the early stage of SARS-CoV-2 infection, resulting in decreased NK cell activity and increased T cell exhaustion.^67–69^ The recovery of NK and CD8+ T cell function was accompanied by the recovery of NKG2A.^70^

## 10. IC LIGANDS

ICs rely on binding with their corresponding ligands for different immune signal ligand expression, the greater the possibility of affecting the occurrence and development of diseases through these pathways.^71,72^ Currently, inhibitors designed to target ligands have been widely used in the treatment of solid tumors eg, PD-L1 inhibitors.^73^ Several studies have also reported the alteration of IC ligands in COVID-19 patients.

## 11. PD-L1

PD-L1 and indoleamine 2,3-dioxygenase were strongly and diffusely expressed in COVID-19 lung parenchymal lesions.^74^ Early studies have found high concentrations of PD-L1 in monocytes/ macrophages and the plasma of BALF.^75^ In addition, PD-L1 expression in basophils and eosinophils correlates with COVID-19 severity.^72^ The expression of PD-L1 gradually increased with the progression of the disease and gradually returned to normal after treatment.^44,45^ Under the stimulation of SARS-CoV-2, the proportion of PD-L1+DC cells increases, indicating that COVID-19 can induce dendritic cells (DCs) to differentiate into an anti-infective phenotype.^76^

## 12. GALECTIN

Galectin plays an important role in the regulation of the immune and inflammatory response. Based on different structures, galectin can be divided into Gal-1, Gal-3, and Gal-9.^77^ All of the Gal proteins can be detected in APC cells (macrophages, monocytes, and DCs) and plasma from COVID-19 patients, which are considered to be associated with “cytokine storm syndrome” and severe disease course.^29^ Interestingly, Gal-9 appears to have higher expression in activated HLA-DR+ and CD80+ monocytes, whereas Gal-9 is down-regulated in activated neutrophils.^78^

In addition, CD155 and CD112 can be detected in APC cells as specific TIGIT ligands on T cells and NK cells. Abnormal expression of CD155, nectin-4, and CD112 has been detected in SARS-CoV-2 infected cells.^48^ These ligands and the opposite ligands, such as DNAM-1, play a role in balancing and maintaining immune cell homeostasis.^48,79^

## 13. THE ROLE OF HIGHER EXPRESSED ICs IN COVID-19

Beyond the understanding that increased IC expression may result on T cell exhaustion in COVID-19 patients, several studies also showed that such T cells have can recognize SARS-CoV-2 peptide and produce associate cytokine. An in vitro antigen co-stimulation study confirmed that SARS-CoV-2 antigen can induce T cells from critical patients to express higher levels of co-inhibitory receptors such as CTLA-4, PD-1, VISTA, and Tim-3, which is related to the production of high levels of TNF-α and IFN-γ.^37^ Moreover, it was reported that CTLA-4 was still highly expressed on CD4+ T cells (including SARS-CoV-2–specific CD4+T cells) 1 year after recovery.^58^ Schultheiss C et al found that COVID-19 patients secreted high concentrations of the inflammatory factors IL-6, IL-10, TNF-α, and TNF-β at the acute stage of infection accompanied by an increase in BTLA expression of CD4+ and CD8+ T cells.^40^ Under the stimulation of SARS-CoV-2, the proportion of PD-L1+DC cells increases, indicating that COVID-19 can induce DCs to differentiate into an anti-infective phenotype.^76^ Thus, the researchers thought that increased IC expression may play potentiate the hyperinflammatory response in COVID-19 patients. At least, it may either play a protective or detrimental role in COVID-19 patients.^37^ Therefore, how to balance the effect of ICs is an open question to the clinicians.

## 14. ICs BLOCKADE IN COVID-19

Based on the understanding that ICs can mediate the immune escape of pathogens and studies of the abnormal expression of ICs on immune cells of patients with COVID-19, ICB may contribute to virus clearance. This idea has attracted the attention of clinical oncologists because IC inhibitors (ICIs) are primarily used for cancer immunotherapy. Patients with cancer in the context of the COVID-19 epidemic have higher infection rates and risk for serious complications.^79^ The clinical response of cancer patients with COVID-19 during ICI treatment is an important reference for guiding ICI use for COVID-19 patients. This is based on the concern that there may be potential synergistic effects for ICB and COVID-19 pathogenesis that leads to aggravated lung injury. However, according to the German working group of a dermato-oncology database, a study involving 13 melanoma patients with COVID-19 demonstrated that only 2 cases developed disease progression and needed hospitalization when using anti-PD-1 antibody alone or in combination with CTLA-4 blocking therapy.^50^ One case was an 83-year-old patient with multiple organ dysfunction who received nivolumab + ipilimumab.^50^ Another case was hospitalized due to fever and diarrhea after nivolumab.^50^ Klebanov et al conducted a retrospective study of 1545 cancer patients who received ICI treatment during the SARS-CoV-2 epidemic.^80^ The results demonstrated that ICI treatment did not increase the risk of COVID-19.^80^ Another report presented data from 41 patients who received PD-1 inhibitors and demonstrated that PD-1 blockade did not increase the risk of progression for COVID-19.^81^ A report from Pala L et al also supported this result where a patient with SARS-CoV-2 and metastatic melanoma was cured by anti-PD-1 therapy.^82^ However, it has been reported that approximately 3 to 5% of patients have an immune-mediated lung injury, which proves that there are synergistic effects between ICB and SARS-CoV-2–related immune pneumonia.^23,83^ Clinical trials are also evaluating the safety and efficacy of PD-1 blockade strategies in COVID-19 patients without cancers (eg, NCT04356508, NCT04335305, NCT04413838, and NCT04343144) (Table 3). Overall, the above data provide the possibility of targeted IC therapy for COVID-19 patients.

**Table 3:** Immune checkpoint blockade in clinical trials for COVID-19 patients.

Trials No.	Condition or disease	Interventions	Primary outcome measures	Secondary outcome measures	Research status
NCT04356508	COVID-19; SARS-CoV-2; pneumonia	Nivolumab + best supportive care	Viral clearance kinetics	(1) Treatment related adverse events of nivolumab (2) Lymphocyte kinetics (3) Cytokine kinetics (4) Length of inpatient stay due to COVID-19. etc	Not yet recruiting
NCT04335305	COVID-19; pneumonia	Tocilizumab plus Pembrolizumab	Percentage of patients with normalization of SpO2 ≥96% on room air	(1) Proportion of patients discharged from the emergency department and classified as low risk (2) Number of days of patient hospitalization (3) Change from baseline in organ failure parameters (4) Incidence of adverse events. etc	Recruiting
NCT04413838	COVID-19	Nivolumab+ nursing routine	Patient’s clinical state	(1) Proportion of death at D7 and D15 (2) Oxygen flow needs (3) Adverse events (4) Discharge from hospital. etc	Not yet recruiting
NCT04343144	COVID-19	Nivolumab	Time to clinical improvement	(1) Overall survival (D28, D90) (2) Cumulative incidence of ICU admission (3) Length of hospital stay (4) Incidence of adverse events. etc	Not yet recruiting

## 15. SUMMARY

Significant alterations in ICs have been characterized in immune cells from patients with COVID-19 or those with SARS-CoV-2 infection, and these interfere the balance of activation and proliferation of various immune cells. These alterations may be associated with disease progression and poor prognosis, particularly in the elderly with more complications. Data from cancer patients with COVID-19 who are undergoing ICI treatment demonstrate that ICB may have defensive functions against COVID-19. However, the possibility of synergistic immune lung injury also needs to be considered when managing and optimizing ICB for COVID-19 patients. Nevertheless, the differences in IC expression in different populations and immune cell subsets and the advantages and disadvantages of IC proteins in different stages of COVID-19 as well as the benefits and risks of ICB in treating different cancers and non-cancer COVID-19 patients still need to be thoroughly research and discussed.
